# Polyphenol-Rich Extracts from *Toona sinensis* Bark and Fruit Ameliorate Free Fatty Acid-Induced Lipogenesis through AMPK and LC3 Pathways

**DOI:** 10.3390/jcm8101664

**Published:** 2019-10-11

**Authors:** Yung-Chia Chen, Hsin-Ju Chen, Bu-Miin Huang, Yu-Chi Chen, Chi-Fen Chang

**Affiliations:** 1 Graduate Institute of Medicine, College of Medicine, Kaohsiung Medical University, Kaohsiung 80708, Taiwan; sshin810512@gmail.com; 2 Department of Anatomy, School of Medicine, College of Medicine, Kaohsiung Medical University, Kaohsiung 80708, Taiwan; 3 Department of Anatomy, College of Medicine, National Cheng Kung University, Tainan 70101, Taiwan; bumiin@mail.ncku.edu.tw; 4 Department of Urology, E-Da Hospital, Kaohsiung 82445, Taiwan; yuchichen1978@gmail.com; 5 Department of Urology, E-Da Cancer Hospital, Kaohsiung 40402, Taiwan; 6 Department of Anatomy, School of Medicine, China Medical University, Taichung 40401, Taiwan; cfchang@mail.cmu.edu.tw

**Keywords:** *Toona sinensis*, lipid accumulation, lipogenesis, LC3, AMPK, HepG2

## Abstract

Non-alcoholic fatty liver disease (NAFLD) is a chronic liver disease found worldwide. The present study aimed to evaluate the mechanisms of inhibiting lipid accumulation in free fatty acid (FFA)-treated HepG2 cells caused by bark and fruit extracts of *Toona sinensis* (TSB and TSF). FFA induced lipid and triglyceride (TG) accumulation, which was attenuated by TSB and TSF. TSB and/or TSF promoted phosphorylation of AMP-activated protein kinase (AMPK) and acetyl-coA carboxylase and peroxisome proliferator-activated receptor alpha upregulation. Furthermore, TSB and TSF suppressed FFA-induced liver X receptor, sterol regulatory element-binding transcription protein 1, fatty acid synthase, and stearoyl-CoA desaturase 1 protein expression. Moreover, TSB and/or TSF induced phosphorylation of Unc-51 like autophagy-activating kinase and microtubule-associated protein 1A/1B-light chain 3 expressions. Therefore, TSB and TSF relieve lipid accumulation by attenuating lipogenic protein expression, activating the AMPK pathway, and upregulating the autophagic flux to enhance lipid metabolism. Moreover, TSB and TSF reduced TG contents, implying the therapeutic use of TSB and TSF in NAFLD.

## 1. Introduction

Non-alcoholic fatty liver disease (NAFLD) is a global health issue driven by dysregulation of lipid homeostasis [1]. NAFLD is a multifactorial disease that is caused not by excessive alcohol consumption but by pathological accumulation of triacylglycerol in hepatocytes [1]. Excessive import or diminished export or oxidation of free fatty acids (FFAs) in hepatocytes causes hepatic steatosis [2]. Hepatic triglyceride (TG) accumulates through glycerol and FFA esterification [2]. For unknown reasons, some patients develop non-alcoholic steatohepatitis and, in more severe cases, liver fibrosis, cirrhosis, and even hepatocarcinoma [2].

NAFLD presents as a metabolic liver disorder [3,4]. With an imbalance of lipid metabolism in the liver, insulin fails to inhibit lipolysis of adipose tissue, increasing the efflux of FFA into the circulation and its uptake by the liver [3]. FFA can be transported into the mitochondrial matrix by specific transport proteins and undergoes β-oxidation [5,6,7,8]. Hyperinsulinemia directly inhibits β-oxidation of FFA, promoting hepatic de novo lipid synthesis through upregulation of sterol regulatory element-binding protein-1c (SREBP-1c) and carbohydrate response element-binding protein [5,6,7,8]. Recent studies have applied in vitro approaches to determine the molecular mechanisms involved in hepatic steatosis progression [9].

Emerging evidence suggests that AMP-activated protein kinase (AMPK), peroxisome proliferator-activated receptor alpha (PPARα) and LC3 are critical regulators of hepatic lipid metabolism and could be therapeutic targets in NAFLD [10]. These target proteins exhibit interactions; for example, phosphorylation of Thr172 in the α-subunit of AMPK activates acetyl-coA carboxylase (ACC)-SREBP-1c, PPARα, and Unc-51-like autophagy activating kinase (ULK1)-LC3 [11,12].

*Toona sinensis* (A. Juss.) M. Roem., a deciduous tree, is widely distributed in Southeast Asia and cultivated in many parts of the world [13]. The whole plant can be used in herbal remedies, and its tender leaves have been used in dishes or sauces for several years [13]. Until now, hundreds of phytochemical compounds have been identified in *T. sinensis*, including polyphenols (phenolic acid, flavonoids, stilbene, and lignan), terpenoids, and sterols [14]. It was reported that aqueous extract of leaves of *T. sinensis* (TSL-1) exhibits many biological functions, such as antiviral [15,16], antibacterial [17], antidiabetic [18], anti-obesity [19], hepatoprotective [20,21], and anti-cancer [22,23,24] functions. However, little is known about other parts of *T. sinensis*. Our preliminary results demonstrated that, among the root, leaves, bark, and fruit of *T. sinensis*, the bark and fruit could block lipid accumulation in hepatocytes. It has been shown that a diet with *T. sinensis* leaves, root, or bark (TSB) enhances sperm quality and improves memory in senescence-accelerated prone-8 mice [25]. *T. sinensis* fruit (TSF) extract exhibits strong antioxidative effects and protects the kidney from diabetic nephropathy [26]. The present study investigated the molecular mechanism of the effects of TSB and TSF extracts on lipid accumulation using an in vitro cellular model.

## 2. Materials and Methods

### 2.1. Chemicals

The 3-(4,5-dimethylthiazol-2-yl)-2,5-diphenyltetrazolium bromide was obtained from GeneMark (GMbiolab Co., Ltd., Taichung, Taiwan). FFA was purchased from Sigma-Aldrich Company (St. Louis, MO, USA). Fenofibrate and chloroquine were obtained from Cayman (Cayman Chemical Co., Ann Arbor, MI, USA). Compound C was obtained from ENZO Life Sciences, Inc. (Farmingdale, NY, USA). Toosendanin was purchased from Wuhan ChemFaces Biochemical Co. Ltd. (Wuhan, Hubei, China).

### 2.2. Herb Authentication

TSF and TSB were collected locally in spring from 2015 to 2018 (Yulin, Taiwan) and identified by Professor Hseng-Kuang Hsu, Physiologist and Botanist, Kaohsiung Medical University, Taiwan.

### 2.3. Preparation of Extracts

The TSB used in the study was obtained from plants aged at least two years, whereas the TSF was collected from a seven-year-old plant. The collected materials were washed and boiled twice with reverse osmosis water for 60 min. Then, the crude extracts were collected to freeze and dry to form powder. TSB and TSF extracts were dissolved in sterile phosphate-buffered saline (PBS; pH 7.4) and filtered using a 0.22-μm syringe filter (Sartorius Stedim Biotech Inc., Göttingen, Germany). 

### 2.4. Experimental Design

To determine the preventive effects of TSB and TSF on lipid accumulation, HepG2 cells were treated with TSB and/or TSF extracts for 24 h. FFA was added to 1% bovine serum albumin (BSA, Sigma) media for another 24 h. The control group was exposed to 1% BSA for the indicated time period. To investigate the AMPK activation, compound C was treated with cells for 30 min, prior to FFA, FFA and TSB, and FFA and TSF co-treatment. To confirm autophagic pathways, TSB and/or TSF were pre-treated with cells for 2 h, prior to 16-h co-treatment with chloroquine.

### 2.5. Cell Culture and Viability Assay

A human hepatoma cell line (HepG2) was purchased from the Bioresource Collection and Research Center (Hsinchu, Taiwan) and grown in Dulbecco’s Modified Eagle Medium (Hyclone, a brand of General Electric Company, Boston, MA, USA) containing 4.5 g/L glucose, 100 units/mL penicillin, 100 µg/mL streptomycin, and 10% foetal bovine serum (Gibco, Grand Island, NY, USA) in a humidified atmosphere with 5% CO_2_ at 37 °C. Cell viability was measured by a quantitative colorimetric assay with 3-(4,5-dimethylthiazol-2-yl)-2,5-diphenyltetrazolium bromide (MTT). After removing the media, MTT solution (0.1 mg/mL) was added to each well for 3 h incubation at 37 °C, and the optical density (OD) was measured at 570 nm with a microplate reader (BioTek Instruments, Inc., Winooski, VT, USA).

### 2.6. Oil Red O Staining

After fixation with formaldehyde, neutral lipids were stained using 0.5% Oil Red O (Bio Basic Inc., Amherst, NY, USA) in isopropanol for 1 min. After removing the staining solution, the OD was measured at 500 nm using a microplate reader (BioTek).

### 2.7. Nile Red Staining

Cells were supplemented with FFA, with or without *T. sinensis* extracts, for 24 h, fixed with 10% formaldehyde and incubated for 10 min with 10 μg/mL Nile red in PBS, and the OD was measured using a multimode microplate reader (BioTek).

### 2.8. TG Assay

TG levels in cell lysates were determined using a colorimetric assay (Cayman Chemical, Ann Arbor, MI, USA) according to the manufacturer’s instructions. After several PBS washes, the scraped cell lysates were centrifuged at 1500 g for 10 min. The cold standard diluent assay buffer was added to resuspend the lysates, and sonication was performed 20 times with 1-s pulse procedure. The lysates were centrifuged at 10,000 g for 10 min, and the pellets were stored in the refrigerator at −80 ^°^C until use.

### 2.9. Gel Electrophoresis and Western Blotting

After treatment, cells in 35-mm dishes were washed with PBS, collected in a lysis buffer (0.15% Triton X-100, 2 mM MgCl_2_, 25 mM HEPES(N-2-Hydroxyethylpiperazine-Nʹ-2-ethanesulfonic Acid), 60 mM PIPES(1,4-Piperazinediethanesulfonic acid), 1 mM EDTA(Ethylenediaminetetraacetic acid), 1 mM phenylmethylsulphonyl fluoride, 1 mM sodium fluoride, 1 mM sodium orthovanadate, 1 mM β-glycerol phosphate, 2.5 mM sodium pyrophosphate, 1 µg/mL aprotinin, 1µg/mL pepstatin A, and 1 µg/mL leupeptin; pH 6.9) and sonicated 20 times with 1-s pulses. Protein levels were measured using a protein assay kit (Bio-Rad Life Sciences, Hercules, CA, USA), and the samples were stored at −80 °C until further analysis.

Protein samples were loaded in lanes (80–160 µg per lane) of a 10–12.5% sodium dodecyl sulphate polyacrylamide gel, subjected to electrophoresis, and transferred to nitrocellulose membranes. Membranes were blocked for 1 h at room temperature with 5% non-fat milk or 5% BSA (for phosphorylated antibodies) in Tris-buffered saline (TBS; 150 mM NaCl, 50 mM Tris base, pH 8.2) containing 0.1% Tween 20 and incubated overnight at 4 °C with the desired primary antibody (Table 1) diluted in TBS, 0.1% Tween 20, and 5% non-fat milk or 5% BSA. Alkaline phosphatase-conjugated anti-rabbit or anti-mouse (1:4000 dilution, GeneTex, Inc., Irvine, CA, USA) and bound antibodies were then detected using an alkaline phosphatase staining system (Sigma).

### 2.10. Liquid Chromatography–Mass Spectrometry (LC–MS)

A Waters ACQUITY UPLC system (Waters Corporation, Milford, MA, USA), coupled with a tandem MS (Finnigan TSQ Quantum Ultra triple-quadrupole MS, Thermo Electron, San Jose, CA, USA), in combination with the Xcalibur software (Thermo-Finnigan, Bellefonte, PA, USA) was used to detect and quantify analytes. The LC–MS system was equipped with an electrospray ion source and ran in negative mode. The injection volume was 10 μL on an ACQUITY UPLC CSH Phenyl-Hexyl column (130 Å, 1.7 µm, 2.1 mm × 100 mm, Waters Corporation, Milford, MA, USA) equipped with a filter (Waters Acquity UPLC™ BEH C18 column, 1.7 μm, 2.1 mm × 5 mm) in front of the column. The flow rate was 300 μL/min, and the column temperature was 40 °C. Solvents were 0.1% acetic acid in water (A) and 0.1% acetic acid in acetonitrile (B).

### 2.11. Total Phenolic Assay

Total phenolics were determined by using the Folin-Ciocalteu method [27]. Briefly, TSB and TSF extracts were sonicated in 80% methanol at room temperature and centrifuged at 14,000 r.p.m. for 5 min. Two microliter sample supernant was mixed with 1.58 mL deionized water and 100 μL Folin reagent was then added into each tube for at least 1 min. Three hundred μL of sodium carbonate solution (20%) was added and mixed thoroughly. After 1 h incubation, the absorbance was read at 765 nm by a multimode microplate reader (Bio-Tek). The total phenolic content was calculated by a equation. Total phenolics = concentration of extract (mg/mL) × volume of extract (mL)/weight of extract (g).

### 2.12. Statistical Analysis

All analyses were performed at least three times, and values are expressed as mean ± standard deviation. Statistical differences were determined by Kruskal–Wallis test and corrected by Wilcoxon signed rank (control vs. FFA) and Mann–Whitney tests (GraphPad Prism software version 7.0, GraphPad Software company, San Diego, CA, USA). A P-value < 0.05 was considered statistically significant.

## 3. Results

### 3.1. Ingredients of Polyphenol Compounds in T. sinensis Extracts

**Figure 1:**
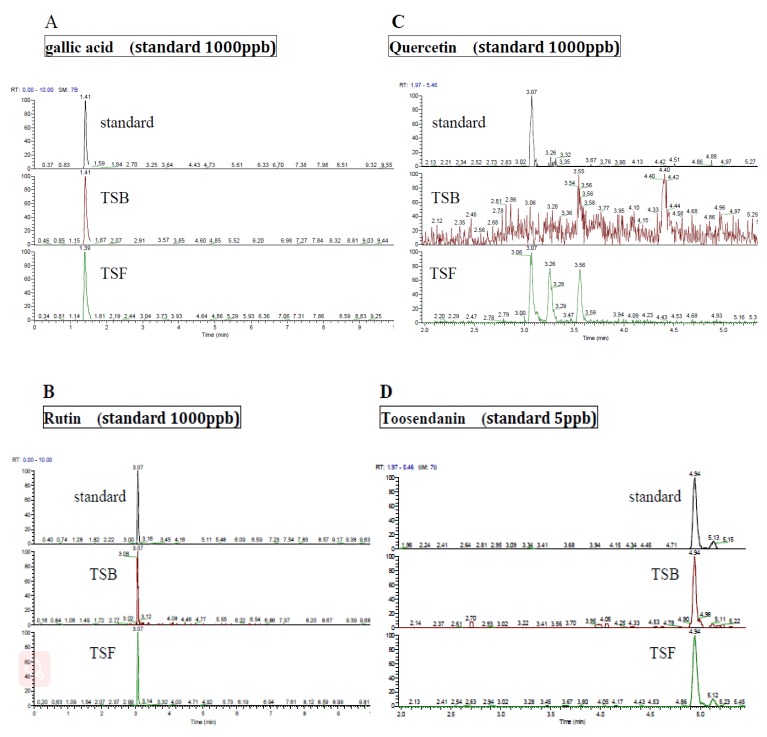
LC–MS analysis of *T. sinensis* leaves, root, or bark (TSB) and *T. sinensis* fruit (TSF) extracts. (**A**) Gallic acid, (**B**) rutin, (**C**) quercetin, and (**D**) toosendanin.

LC-MS has been used to determine the levels of the possible active compounds in TSB and TSF extracts. Gallic acid was the most abundant ingredient in TSB (300400 ppb) and TSF (1424800 ppb extracts (Figure 1A). Rutin can be found in both TSB (142 ppb) and TSF (3015 ppb) extracts (Figure 1B). Quercetin was noted only in TSF extract (4088 ppb) (Figure 1C). Although kaempferol inhibited FFA-induced lipid accumulation, it is not present in the two extracts. Toosendanin is exhibited in an extremely low amount in both TSB (0.8 ppb) and TSF extracts (3.8 ppb) (Figure 1D). The retention time of each compound is 1.47 min for gallic acid, 3.07 min for rutin and quercetin and 4.94 min for toosendanin. The amount of total phenolics are 42 mg/g gallic acid equivalent in TSB and 44 mg/g gallic acid equivalent in TSF, respectively (Table S1).

### 3.2. FFA Induces Lipid Accumulation in HepG2 Cells

**Figure 2:**
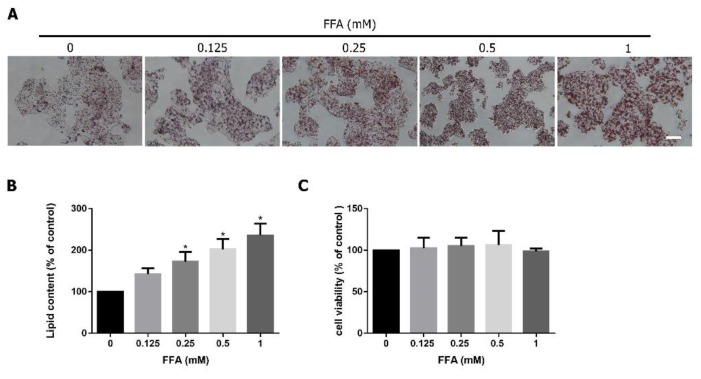
Effects of free fatty acids (FFAs) mixture on lipid accumulation and cytotoxicity in HepG2 cells. (**A**) Oil Red O staining. (**B**) Quantification of lipid contents. (**C**) MTT assays. The representative images are from six independent experiments. Scale bar: 50 μm. Data were quantified for three to six independent experiments and expressed as mean ± SD. * *P* < 0.05 compared with the control group (0 mM).

The human hepatoma cell line HepG2 was used to mimic FFA-induced steatosis in the human body [9]. Oleic and palmitic acids are the most abundant FFAs in patients with steatosis [28]. A lipid-laden HepG2 cell model was established by adding different FFA levels (oleic acid 0.66 mM, palmitic acid 0.33 mM) for 24 h. As shown in Figure 1, FFA induced lipid accumulation in a concentration-dependent manner (Figure 2A,B) without cytotoxicity (Figure 2C). FFA (1 mM) induced about twofold lipid accumulation in HepG2 cells based on Oil Red O staining.

### 3.3. TSB and TSF Extracts Inhibit Lipid Accumulation in Lipid-Laden HepG2 Cells

**Figure 3:**
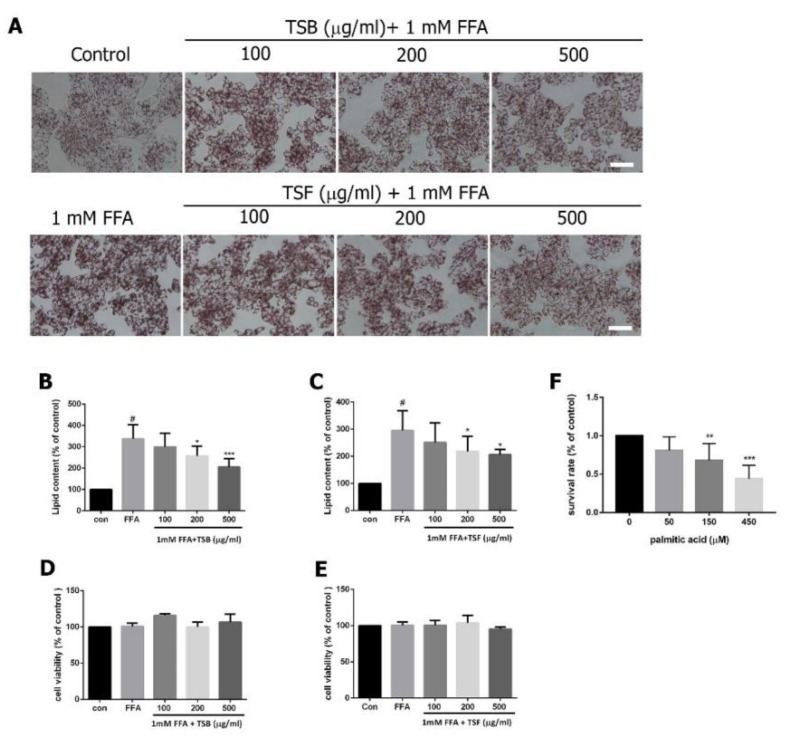
Effects of TSB and TSF extracts on lipid accumulation and cytotoxicity in HepG2 cells. (**A**) Oil Red O staining. Scale bar = 50 μm. The representative images are from eight independent experiments. (**B** and **C**) Quantification of lipid contents. (**D**–**F**) MTT assays. Data were quantified for three to twelve independent experiments and expressed as mean ± SD. ^#^ *P* < 0.05 compared with the control group (0 g/mL). * *P* < 0.05 compared with the FFA group. In B–D, *** *P* < 0.001. In Figure 3F, ** *P* < 0.01, *** *P* < 0.001 compared with contro (0 μM).

**Figure 4:**
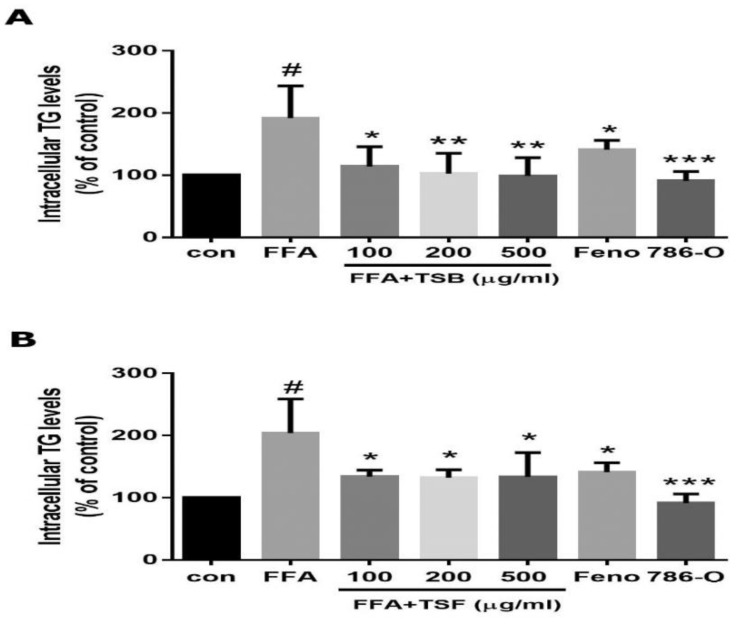
Effects of TSB (**A**) and TSF (**B**) extracts on FFA-induced intracellular triglyceride levels. Fenofibrate (Feno, 125 μM) was used as a positive control. In renal carcinoma cells, 786-O served as a negative control. The bar graphs show the quantification of the triglyceride contents. Data from six independent experiments are expressed as mean ± SD. ^#^ *P* < 0.05 compared with the control group (con). * *P* < 0.05, ** *P* < 0.01, *** *P* < 0.001 compared with the FFA group. (please add the sentence after * *P* < 0.05.

To determine TSB and TSF’s inhibitory effects on FFA-induced lipogenesis in HepG2, TSB and TSF extracts were pre-treated with cells for 24 h. FFA was added to the cells for another 24 h. Oil Red O and Nile red staining confirmed that TSB and TSF reduced FFA-induced lipogenesis in a concentration-dependent manner (Figure 3A–C). In the TSB-treated group, the lipid content was reduced by 11%, 23%, and 39% at levels of 100, 200, and 500 μg/mL when compared with those in the FFA group (Figure 3A upper panel and 3B). In the TSF-treated group, the lipid content was reduced by 15%, 26%, and 30% at levels of 100, 200, and 500 μg/mL when compared with those in the FFA group (Figure 3A lower panel and 3C). Co-treatment with TSB and FFA, or TSF and FFA, did not affect the cells’ survival rates (Figure 3D,E), whereas palmitic acid (150 and 450 μM) alone showed cytotoxicity to HepG2 cells (Figure 3F). To further investigate the anti-lipogenic effects in FFA-treated HepG2 cells, the TG levels were examined under the same experimental conditions. As shown in Figure 4, both TSB and TSF significantly inhibited TG expression in cells (Figure 4A,B). In renal carcinoma cells, 786-O was used as the negative control, while fenofibrate was the positive control. 

### 3.4. TSB and TSF Extracts Reduce Lipogenesis

**Figure 5:**
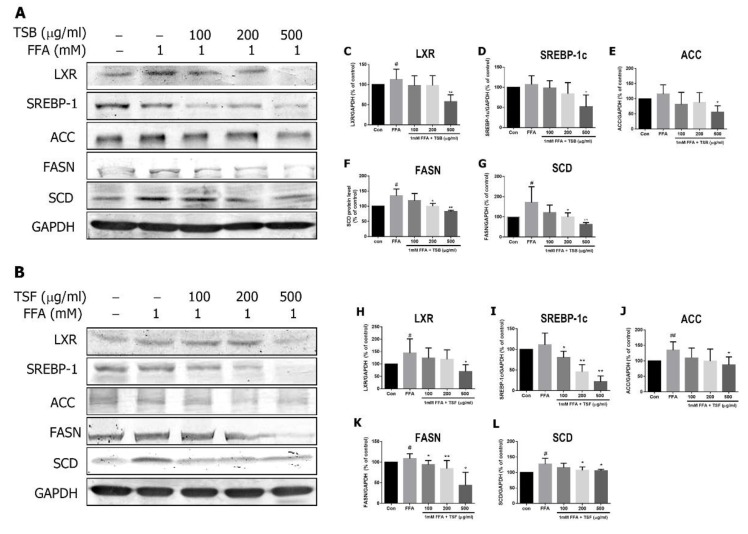
Effects of TSB and TSF extracts on FFA-induced lipogenic protein expression. (**A** and **B**) Immunoblots of lipogenic protein expression in TSB and TSF pre-treated lipid-laden HepG2 cells. GAPDH was used as the internal control. (**C**–**L**) The bar graphs show densitometric data (mean ± SD) from three to eight independent experiments. The images shown represent one experiment. ^#^ *P* < 0.05 and ^##^ P < 0.01 compared with the control group (con). * *P* < 0.05, ** *P* < 0.01 and *** *P* < 0.001 compared with the FFA group.

To study TSB and TSF’s mechanisms on FFA-induced lipid accumulation, cells were pre-treated with TSB and/or TSF extracts and examined for the related lipogenic protein expression, including liver X receptor (LXR), SREBP-1c, ACC, fatty acid synthase (FASN), and stearoyl-CoA desaturase 1 (SCD1). TSB and TSF extracts significantly inhibited FFA-induced LXR, SREBP-1, ACC, FASN, and SCD1 expression (Figure 5A–L).

### 3.5. TSB and TSF Extracts Regulate Lipid Metabolism through the AMPK Pathway

**Figure 6:**
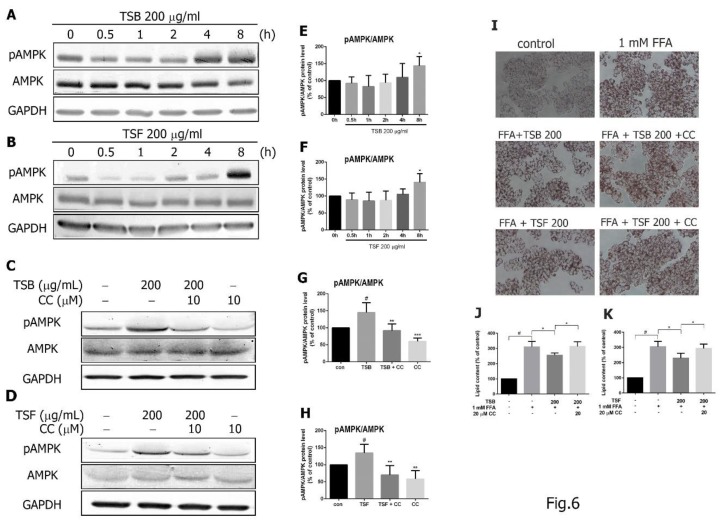
TSB and TSF extracts’ effects on AMPK activation in HepG2 cells. (**A**–**D**) Immunoblots of phosphorylated AMPK expression in HepG2 cells. Total AMPK and GAPDH were used as the internal control. (**E**–**H**) The bar graphs show densitometric data (mean ± SD) from three to ten independent experiments. (**I**) Oil Red O staining. (**J** and **K**) Quantification of lipid contents. ^#^ *P* < 0.05 compared with the control group (0 h). * *P* < 0.05 compared with the FFA group. ** *P* < 0.01 and *** *P* < 0.001 compared with the FFA group. Scale bar = 50 μm.

To determine whether TSB and TSF extracts inhibit lipid accumulation by activating the AMPK pathway, cells were treated with TSB and/or TSF extracts for 0, 0.5, 1, 2, 4, and 8 h, and phosphorylation of AMPK (Thr172) was determined by Western blotting. TSB and TSF extracts significantly increased AMPK phosphorylation at 8 h (Figure 6A,B,E,F). Additionally, TSB and TSF extract-induced AMPK phosphorylation was reduced by treatment with AMPK inhibitor compound C (Figure 6C,D,G,H). To evaluate whether AMPK phosphorylation mediates the TSB and TSF’s effects on lipid accumulation in FFA-treated hepatocytes, HepG2 cells were treated with compound C 30 min prior to treatment with *T. sinensis* extracts. Lipid accumulation was measured 24 h after FFA exposure using Oil Red O staining. At a level of 200 μg/mL, both TSB and TSF significantly inhibited lipid accumulation in FFA-treated HepG2 cells, but these effects were reversed by compound C (Figure 6I–K).

### 3.6. TSB and TSF Extracts Regulate Lipid Metabolism through Activation of AMPK-ACC and PPARα Pathways

**Figure 7:**
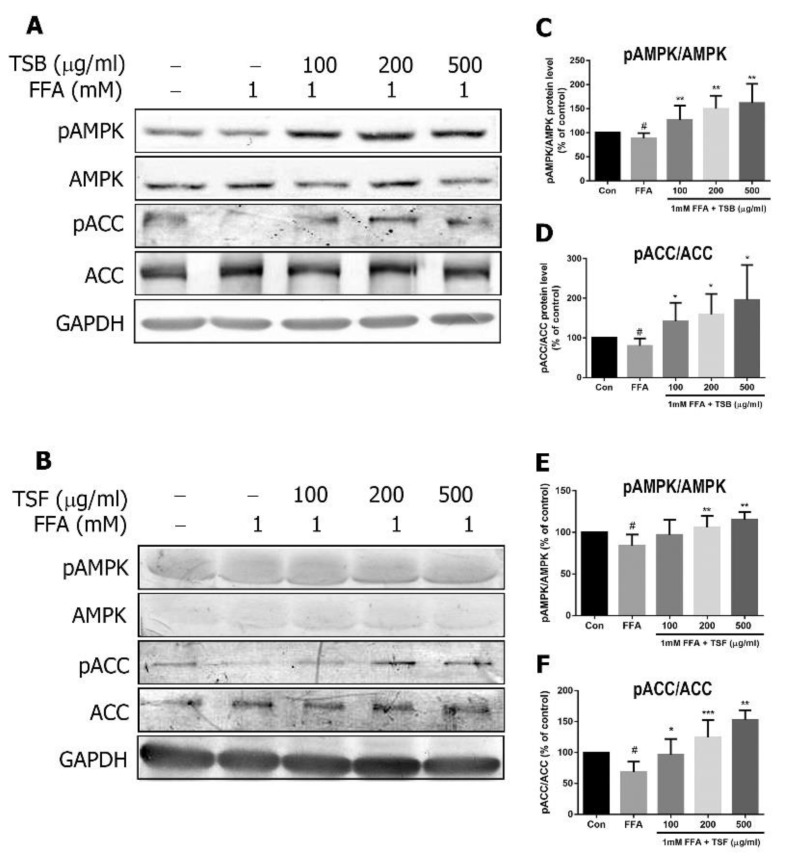
TSB and TSF’s effects on phosphorylated AMPK and ACC expression in FFA-treated HepG2 cells. (**A** and **B**) Immunoblots of phosphorylated AMPK and ACC levels. AMPK, ACC and GAPDH were used as internal controls. (**C**–**F**) The bar graphs show densitometric data (mean ± SD) from three to ten independent experiments. ^#^ *P* < 0.05 compared with the control group (con). * *P* < 0.05 and ** *P* < 0.01 compared with the FFA group. *** *P* < 0.001 compared with the FFA group.

**Figure 8:**
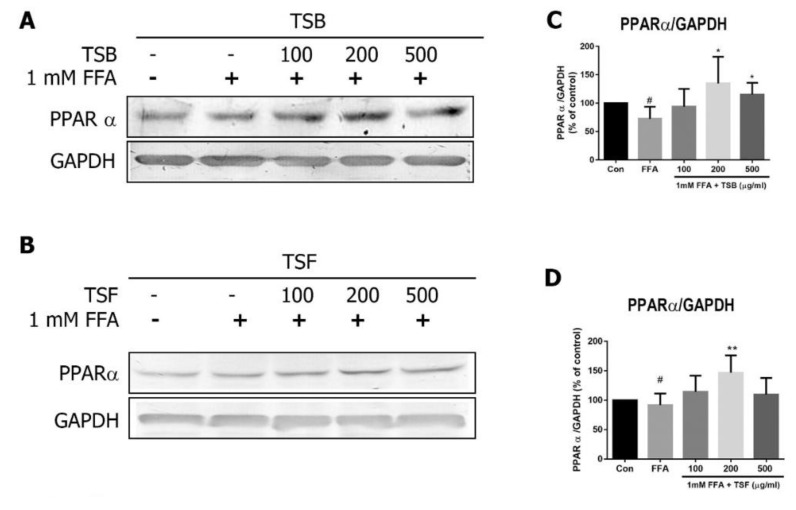
TSB and TSF’s effects on PPARα expression in FFA-treated HepG2 cells. (**A** and **B**) Immunoblots of PPAR. (**C** and **D**) The bar graphs show densitometric data (mean ± SD) from three to five independent experiments. ^#^ *P* < 0.05 compared with the control group (con). * *P* < 0.05 and ** *P* < 0.01 compared with the FFA group.

Once the AMPK pathway is activated, it can directly promote downstream effectors, including ACC phosphorylation and PPAR expression [29]. Compared with the control group, the FFA group had decreased AMPK and ACC phosphorylation levels (Figure 7A–F). After TSB and/or TSF treatment, the phosphorylation levels of AMPK and ACC were enhanced in the FFA-treated group (Figure 7A–F). Similarly, PPAR expression was downregulated by FFA treatment but upregulated by TSB and/or TSF treatment (Figure 8A–D).

### 3.7. TSB and TSF Extracts Induce Autophagic Flux to Decrease Lipid Accumulation in Lipid-Laden HepG2 Cells

**Figure 9:**
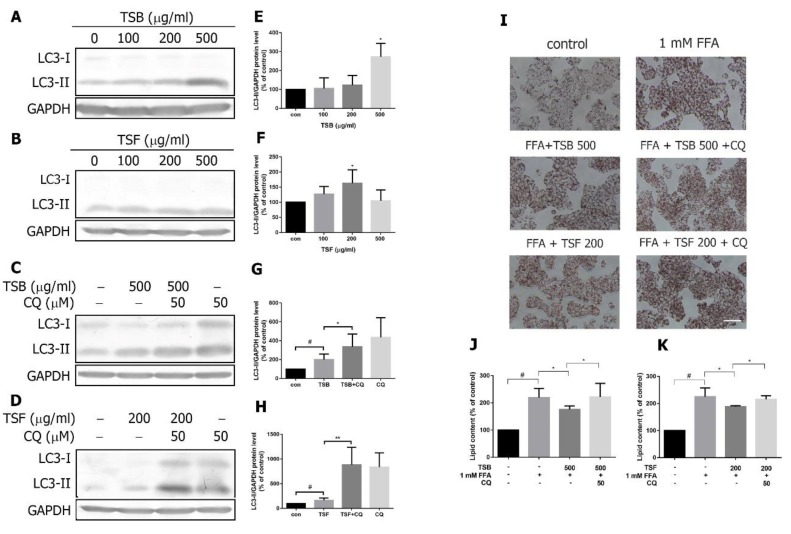
Effects of TSB and TSF on autophagic flux in HepG2 cells. (**A**–**D**) Immunoblots of LC3 subunits. (**E**–**H**) The bar graphs show densitometric data (mean ± SD) from three to five independent experiments. (**I**) Oil Red O staining. (**J** and **K**) Quantification of lipid contents. ^#^ *P* < 0.05 compared with the control group (con). * *P* < 0.05 and ** *P* < 0.01 compared with the FFA group. Scale bar = 50 μm.

NAFLD was related to dysregulation of the autophagic process in hepatocytes [30]. To investigate the TSB and TSF extracts’ effects on autophagy, HepG2 cells were examined to determine whether TSB and/or TSF extracts induced LC3 expression. After the 24 h treatment, TSB (500 μg/mL) and TSF (200 μg/mL) extracts significantly induced LC3-II expression (Figure 9A,B,E,F). Moreover, LC3-II expression was largely upregulated by adding the autophagosome inhibitor chloroquine (50 μM) to TSB- and TSF-treated cells (Figure 9C,D,G,H). To determine whether TSB and TSF extract-induced autophagy was associated with decreased lipid content, HepG2 cells were pre-treated with TSB and/or TSF extracts for 2 h and co-treated with chloroquine (50 μM) and FFA for further 16 h. Lipid accumulation was measured by Oil Red O staining. Both TSB and TSF extracts inhibited FFA-induced lipid accumulation, but these effects were blocked by chloroquine (Figure 9I–K).

### 3.8. TSB and TSF Extracts Regulate Lipid Metabolism through AMPK Downstream ULK1 and LC3 Pathways

**Figure 10:**
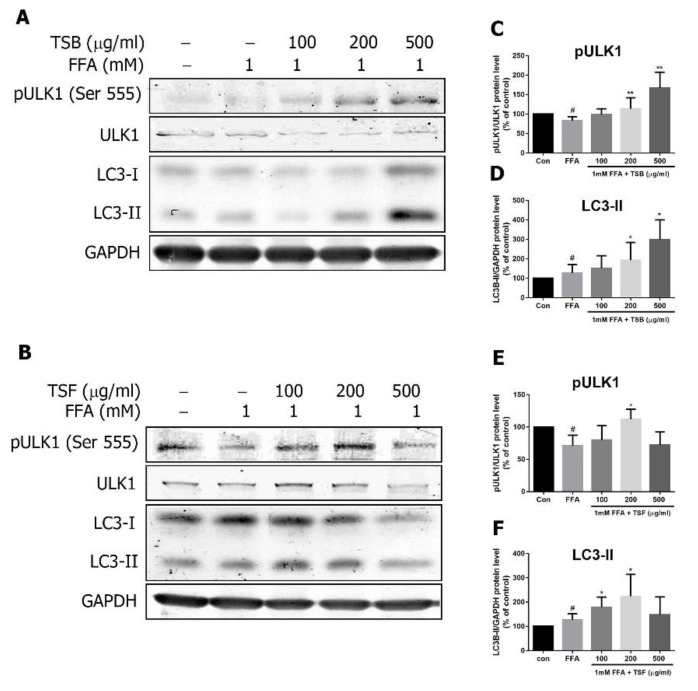
Effects of TSB and TSF on the LC3 pathway in FFA-treated HepG2 cells. (**A** and **B**) Immunoblots of phosphorylated ULK and LC3 subunits. (**C**–**F**) The bar graphs show densitometric data (mean ± SD) from three to five independent experiments. ^#^ *P* < 0.05 compared with the control group (con). * *P* < 0.05 and ** *P* < 0.01 compared with the FFA group.

AMPK directly regulates autophagy by phosphorylating, and thereby activating, ULK1 [29]. Four phosphorylation sites within ULK1 (Ser467, Ser555, Thr574 and Ser637) have been mapped [11]. The phosphorylation site Ser555 of ULK1 was reported to be activated by AMPK [11]. Compared with the control, FFA inhibited ULK1 phosphorylation (Figure 10A–F). At a level of 200 μg/mL, TSB and/or TSF extracts significantly enhanced phosphorylation of ULK1 in FFA-treated HepG2 cells, which was accompanied by LC3-II upregulation (Figure 10A–F).

### 3.9. Inhibitory Effects of Compounds from T. sinensis on Lipid Accumulation in FFA-Treated HepG2 Cells

**Figure 11:**
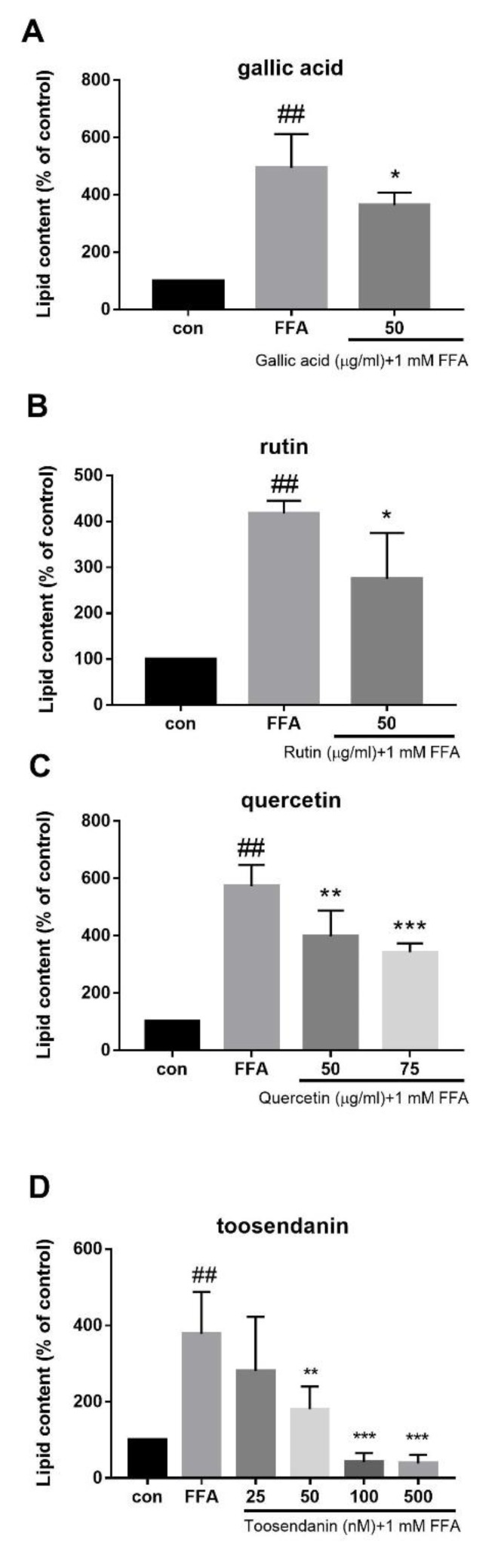
Effects of compounds isolated from *T. sinensis* on lipid accumulation in HepG2 cells. (**A**–**D**) Nile red staining. Data were quantified for three to eight independent experiments and expressed as mean ± SD. ^##^ *P* < 0.01 compared with the control group (con). * *P* < 0.05 and ** *P* < 0.01 compared with the FFA group.

To determine which of *T. sinensis’* active components regulate lipid metabolism, cells were pre-treated with gallic acid, quercetin, rutin, methyl gallate, ethyl gallate, kaempferol, or toosendanin prior to FFA treatment. Lipid accumulation was measured 24 h after FFA exposure by Nile red staining. The effective levels of compounds were nontoxic to all cells. We found that gallic acid, rutin, and quercetin at a level of 50 μg/mL significantly reduced FFA-induced lipid contents (Figure 11A–C), whereas ethyl gallate, methyl gallate, and tannic acid at nontoxic levels had no effects. Additionally, toosendanin at levels of 0.05, 0.1, and 0.5 μM significantly reduced FFA-induced lipogenesis (Figure 11D). 

## 4. Discussion

The incidence of NAFLD varies at a range of 30%–60% worldwide, and the signs are undetectable in the early stages [1]. Currently, effective NAFLD treatment methods include exercise and diet regulation [31]. However, a significant proportion of NAFLD patients do not follow these recommendations. NAFLD is not a benign disease [31]; a substantial proportion of the population is at risk of progressive disease [2]. *T. sinensis* is a popular dish in Asia and Taiwan, and vegetarians use it as flavoring. Since it is nontoxic and easily cultivated, *T. sinensis* is widely used as an herbal remedy [32,33]. TSB and TSF have also been used in traditional medicine [13]. In this study, we found that both TSB and TSF extracts blocked the increase of intracellular TG levels and lipid droplets in FFA-treated hepatocytes. Fatty acids transported to the liver are esterified to TG or oxidised to produce energy [34]. However, an increase in TG level is harmful to hepatocytes [35]. The levels of TSB and TSF extracts used in the study were nontoxic to cells. Interestingly, TSB was even more effective than fenofibrate (a hypolipidaemic PPARα agonist). The anti-steatotic effects of TSB and TSF on NAFLD in an in vivo model are currently being examined.

Several pathways were activated by TSB and/or TSF extracts, including AMPK, PPAR and LC3 expression. AMPK has various physiological functions, such as regulation of energy homeostasis, autophagy, cell polarity, and cell proliferation [12,36]. Previously, AMPK was considered as a potential target in NAFLD treatment [12], by activating downstream target protein PPARwhile inhibiting SREBP-1 through LXR [36,37]. LXRs’ lipogenic activity arises from upregulation of the master regulator SREBP-1c and induction of FASN, ACC and SCD1 [38,39]. Our data are consistent with this finding. TSB and TSF extracts activated AMPK activity while inhibiting FASN, ACC, SCD1, and LXR expression and TG accumulation. Our data showed that inhibition of AMPK activity by compound C deteriorated lipogenesis, suggesting that activation of AMPK by TSB or TSF is indispensable for improving lipid accumulation in FFA-treated HepG2 cells.

It was demonstrated that decreased autophagy in the liver with ageing is associated with hepatic lipid accumulation [30]. ULK1 is a mammalian homologue of Atg1, which is the most upstream component of the autophagy pathway [11]. As the autophagy-initiating kinase, ULK can be directly phosphorylated by AMPK at six different sites during nutrient deprivation [11,40]. TSB and/or TSF extracts induced autophagic flux accompanied by ULK1 phosphorylation at Ser555. Accordingly, inhibition of autophagosome formation by chloroquine significantly increased lipid accumulation in FFA-treated HepG2 cells. These results imply that TSB or TSF regulated lipid metabolism through autophagic mechanism. Thus, the upstream key regulator, which includes mammalian target of rapamycin and Akt, will be examined further.

*T. sinensis* comprise numerous phytochemical components, including gallic acid, methyl gallate, ethyl gallate and rutin, quercetin, quercitrin, kaempferol, catechin, terpenoids, and tannins [41,42]. The present study indicates that TSB and TSF extracts attenuated hepatocellular lipid accumulation and that gallic acid, quercetin, rutin, and toosendanin are active components of the anti-steatotic effects of *T. sinensis* extracts. It has been reported that gallic acid, quercetin, and rutin exhibited anti-steatotic effects on high-fat-diet-fed mice [43,44]. Toosendanin, a triterpenoid extracted from Meliaceae plants, is also known as the major ingredient in Meliaceae fruit, which has been reported to possess antiadipogenic activity in 3T3-L1 preadipocytes [45]. Interestingly, toosendanin in the nanomolar range completely inhibited lipid accumulation, while other compounds attenuated lipid accumulation by only 5%–10%. We suggest that TSB and TSF extracts were complex, and all ingredients in the extracts contributed partly to attenuate lipid accumulation in hepatocytes. Therefore, polyphenol-rich TSB and/or TSF may be a safe and potential herbal remedy to prevent NAFLD.

## 5. Conclusions

Briefly, TSB and TSF extracts do not cause cytotoxicity, suggesting the potential in targeting steatosis. Either TSB or TSB extract shows anti-lipogenic activity and improves lipid metabolism in hepatocytes. Both mainly ameliorate lipid accumulation through AMPK, PPAR, and LC3 pathway activation and inhibit lipogenic protein expression.

## Figures and Tables

**Table 1:** List of antibodies.

Antibody	Dilution	Brand
FASN, *p*-AMPK (Thr 172), AMPK, pACC (Ser79), ACC	1:1000	Cell Signaling Technology Inc. (Danvers, MA, USA)
SREBP-1c, PPARα, SCD1	1:1000	Santa Cruz Biotechnology, Inc. (Santa Cruz, CA, USA)
LXR GAPDH	1:1000 1:3000	Proteintech Group Inc. (Rosemont, IL, USA)
LC3B	1:1000	Novus Biologicals, LLC., a Bio-Techne brand (Centennial, CO, USA)

## Supplementary Materials

The following are available online at https://www.mdpi.com/2077-0383/8/10/1664/s1, Table S1: Total phenol content.
