# Supplementary material for: Polyphenol-Rich Extracts from Toona sinensis Bark and Fruit Ameliorate Free Fatty Acid-Induced Lipogenesis through AMPK and LC3 Pathways
Source: J Clin Med. 2019 Oct 11;8(10):1664. doi: 10.3390/jcm8101664 (PMC6832244; doi:10.3390/jcm8101664)
Supplement: Supplementary file 1 [file jcm-08-01664-s001.pdf]

enago — Author First, Quality First — www.enago.tw | ulatus — localize to globalize — www.ulatus.tw | voxtab — we are all ears — www.voxtab.com

CERTIFICATE OF EDITING

This is to certify that the paper titled **Polyphenol-rich extracts from Toona sinensis bark and fruit ameliorate free fatty acid-induced lipogenesis through AMPK and LC3 pathways** commissioned to us by **Yung-Chia Chen (高醫大)** has been edited for English language, grammar, punctuation, and spelling by Enago, the editing brand of Crimson Interactive Consulting Co. Ltd. under Normal Editing.

✔ **ISO 17100:2015**
Translation Service Providers

✔ **ISO 27001:2013**
Information Security Management System

✔ **ISO 9001:2015**
Quality Management System

Issued by:
Enago, Crimson Interactive Inc.
160, Greentree Dr, Ste 101 street,
Dover City, Kent, Delaware, 19904
Phone: +1-877-712-2177

Disclaimer : The author is free to accept or reject our changes in the document after our editing. However, we do not bear responsibility for revisions made to the document after our edit on **26 Sep 2019**.

Japan www.enago.jp, www.ulatus.jp, www.voxtab.jp
Taiwan www.enago.tw, www.ulatus.tw
China www.enago.cn, www.ulatus.cn
Brazil www.enago.com.br, www.ulatus.com.br
Germany www.enago.de
Russia www.enago.ru
Arabic www.enago.ae
Turkey www.enago.com.tr
S. Korea www.enago.co.kr
Global www.enago.com, www.ulatus.com, www.voxtab.com

About Crimson:
Crimson Interactive INC is one of the world's leading academic research support services. Since 2005, we've supported over 2 million researchers in 125 countries with their publication goals.

Supplementary table 1

Total phenols content

	mg/g gallic acid equivalent
TSB	42.037 ± 1.1315
TSF	44.012 ± 0.523

Data expressed as mean ± S.D. of three samples in triplicates analysed separately.
